# Supplementary material for: Using Non-Violent Discipline Tools: Evidence Suggesting the Importance of Attunement
Source: Int J Environ Res Public Health. 2023 Dec 15;20(24):7187. doi: 10.3390/ijerph20247187 (PMC10742770; doi:10.3390/ijerph20247187)
Supplement: Supplementary file 1 [file ijerph-20-07187-s001.zip › File S1 - Summary of method and search strategy.pdf]

Supplement 1: Summary of method and search strategy

Table of contents

Introduction 2
Method 2
Search terms 3
Databases 3
Inclusion and exclusion criteria 3
Search process and screening 4
Data extraction and management 4
Assessment of methodological quality of included reviews 5
Table S1. *Checklist for review inclusion* 5
Table S2. *Final list of search terms* 5
References 9

Introduction

This paper is the second of two on non-violent discipline. The first paper [1] reported on a systematic overview of evidence on non-violent discipline tools. The current paper explores evidence relating to attunement found in that overview. Full description of the method, intensive search process and results of the overview can be found in the first paper [1], but to facilitate better understanding of where the information for this second paper was obtained, a summary follows.

Method

Information from a very large number of relevant primary studies needed to be found and assessed to meet the goals of the first paper, of finding and exploring the state of the science on non-violent discipline tools. The most suitable research method was therefore the systematic overview. Systematic overviews, also called meta-reviews or umbrella reviews, are similar in method to systematic reviews, but review systematic reviews rather than primary studies.

Designed to create a "friendly front end" to available reviews, overviews make evidence from multiple systematic reviews easily accessible in one document [2] (p. 608). They can cover a much broader field and answer broader questions than a primary study or systematic review, and can integrate information on multiple interventions. They show gaps where more reviews are needed, and can synthesize large amounts of evidence. These attributes make overviews particularly useful for policy makers and others needing to make evidence-informed decisions [2,3,4,5]. The protocol for this overview was based on the approach taken by the Cochrane Handbook for Systematic Reviews of Interventions [2], and approved by a review committee in the Department of Psychology, University of Cape Town.

Search terms

A list of search terms can be found below.

Databases

The following databases were searched: Academic Search Premier; Africa-Wide information; CINAHL; Communication and Mass Media Complete; ERIC; Health Source: Nursing / Academic Edition; Humanities International Complete; Master FILE Premier; MEDLINE; PsycARTICLES; PsycINFO; SocINDEX; Teacher Reference Centre; The Cochrane and Campbell libraries; Education Database.

Inclusion and exclusion criteria

Inclusion criteria for the overview were that systematic reviews had to be published in English, in a peer reviewed journal in 1999 or later, present evidence on at least one non-violent discipline tool, and describe participants of reviewed studies as children, adolescents, juveniles, age 18 and under, or school-going. Where there was a mixture of adult and child participants, the results had to be differentiated, or age had to be tested and found not to moderate results. Reviews had to include behavioral outcomes, or outcomes clearly related to child behavior (e.g. impact of child behavior changes on caregivers). Studies reviewed could include participants, with and without disabilities or medical conditions. Target behaviors could be negative (undesirable) or positive, such as on-task or prosocial behavior, and range from mild to extremely challenging.

Exclusion criteria were: grey literature; violent or coercive interventions; purely architectural environmental interventions or reviews with purely academic or non-behavioral outcomes. Since the focus was on adult-child interactions, reviews of peer interventions, such as

peer mediation and peer tutoring were excluded. The exception to this was adult involvement of peers in modelling a behavior.

In terms of quality, the inclusion criteria were that a review had to be systematic (i.e. describe the search strategy and inclusion criteria for the studies reviewed), and included primary studies had to be clearly referenced. If poor quality reviews met inclusion criteria and were not overlapping, they were included, in order to give a more complete picture of the evidence on each intervention [6]. The list of included reviews can be found in online supplement 3.

Search process and screening

All searches and screening were run independently by the first author and an assistant reviewer. Results were cross-checked and a consensus process followed, occasionally involving the second author. The inclusion checklist used for screening can be found below. Reference lists of all included reviews were searched independently by the first author and an assistant reviewer, and relevant reviews were retrieved and screened following the same consensus process. A PRISMA flow diagram summarizing the overview process can be found in online supplement 2.

Data extraction and management

Data were independently extracted from each review by the first author and an assistant reviewer according to a predetermined extraction protocol. Both reviewers extracted a list of interventions covered by the review, scored the review for quality, and extracted relevant references from the reference list for screening. A consensus process was followed, with any areas of uncertainty resolved in discussion with the second author. The first author completed a more detailed extraction of all other information required for the first paper (such as demographic information, target behaviors and outcomes), as well as the data on attunement, relevant to this paper.

Assessment of methodological quality of included reviews

All included reviews were independently scored by 2 reviewers, using the AMSTAR checklist. A consensus process was followed, involving a third person if needed. A table showing scoring for each AMSTAR question, for all reviews relevant to this paper can be found in the Supplementary Materials S3.

Table S1. *Checklist for review inclusion*

Is it peer reviewed?
Is it in English?
Does it review evidence on children age 18 and under and / or their caregivers (i.e. parents, teachers or other caregivers)? More specifically: 0-18 or school going or juveniles. Reviews that include adults must have differentiated results for children.
Does it review evidence on at least one positive discipline intervention?
Does it present or review any child or caregiver outcomes?

Table S2. *Final list of search terms*

1	SU “listen*” AND (“systematic review” OR “meta-analy*”) NOT “music*”
2	“reflective listening” AND (“review” OR “meta-analy*”)
3	“Communication skill*” AND (“disciplin*” OR “behavio*”) AND (“parent*” OR “famil*” OR “classroom*” OR “school*” OR “child*” OR “adolescen*” OR “youth” OR “teen*” OR “student*”) AND (“systematic review” OR “meta-analy*”)
4	“parent-child communication” AND (“systematic review” OR “meta-analy*”)
5	“emotion coaching” AND (“review” OR “meta-analy*”)
6	(“OTR” OR “opportunit* to respond”) AND (“parent*” OR “famil*” OR “classroom*” OR “school*” OR “child*” OR “adolescen*” OR “teen*” OR “youth” OR “student*”) AND (“review” OR “meta-analy*”)
7	“self-monitor*” AND (“disciplin*” OR “behavio*”) AND (“parent*” OR “famil*” OR “classroom*” OR “school*” OR “child*” OR “adolescen*” OR “teen*” OR “youth” OR “student*”) AND (“systematic review” or “meta-analy*”)
8	“self-management” AND (“disciplin*” OR “behavio*”) AND (“parent*” OR “famil*” OR “classroom*” OR “school*” OR “child*” OR “adolescen*” OR “teen*” OR “youth” OR “student*”) AND (“systematic review” or “meta-analy*”)
9	(“CICO” OR “check in check out”) AND (“review” OR “meta-analy*”)

10	SU (“monitor*” AND (“parent*” OR “teacher*”)) AND (“review” OR “meta-analy*”)
11	“Daily Progress Report” AND (“review” OR “meta-analy*”)
12	SU “antecedent*” AND (“review” OR “meta-analy*”) AND (“parent*” OR “famil*” OR “classroom*” OR “school*” OR “child*” OR “adolescen*” OR “teen*” OR “youth” OR “student*”)
13	SU “choice*” AND (“disciplin*” OR “behavio*”) AND (“parent*” OR “famil*” OR “classroom*” OR “school*” OR “child*” OR “adolescen*” OR “teen*” OR “youth” OR “student*”) AND (“systematic review” OR “meta-analy*”)
14	“choice making” AND (“disciplin*” OR “behavio*”) AND (“parent*” OR “famil*” OR “classroom*” OR “school*” OR “child*” OR “adolescen*” OR “teen*” OR “youth” OR “student*”) AND (“review” OR “meta-analy*”)
15	“response blocking” AND “behavio*” AND (“review” OR “meta-analy*”)
16	“response interruption” AND (“review” OR “meta-analy*”)
17	“response deprivation” AND (“review” or “meta-analy*”)
18	SU “restraint*” AND (“aggressi*” OR “crisis” OR “disciplin*” OR “behavio*”) AND (“parent*” OR “famil*” OR “classroom*” OR “school*” OR “child*” OR “adolescen*” OR “youth” OR “teen*” OR “student*”) AND (“systematic review” OR “meta-analy*”)
19	“Functional Communication Training” AND (“review” OR “meta-analy*”)
20	“replacement behavio*” AND (“review” OR “meta-analy*”)
21	(“high probability request sequence” OR “high probability instruction* sequence”) AND (“review” OR “meta-analy*”)
22	“behavioral momentum” AND (“review” OR “meta-analy*”)
23	“errorless compliance training” AND (“review” OR “meta-analy*”)
24	video (“modeling” OR “modelling”) AND (“systematic review” OR meta-analy*)
25	SU("modeling" OR “modelling”) AND (“parent*” OR “famil*” OR “classroom*” OR “school*” OR “child*” OR “adolescen*” OR “teen*” OR “youth” OR “student*”) AND (“systematic review” OR “meta-analy*”)
26	SU “prompt*” AND (“disciplin*” OR “behavio*”) AND (“parent*” OR “famil*” OR “classroom*” OR “school*” OR “child*” OR “adolescen*” OR “teen*” OR “youth” OR “student*”) AND (“review” OR “meta-analy*”)
27	SU “preparation” AND (“parent*” OR “famil*” OR “classroom*” OR “school*” OR “child*” OR “adolescen*” OR “teen*” OR “youth” OR “student*”) AND (“systematic review” OR “meta-analy*”)
28	SU “priming” AND “behavio*” AND (“parent*” OR “famil*” OR “classroom*” OR “school*” OR “child*” OR “adolescen*” OR ”youth” OR “teen*” OR “student*”) AND (“review” OR “meta-analy*”)
29	“pre-exposure” AND (“review” OR “meta-analy*”) AND (“child*” OR “adolescen*” OR “youth” OR “teen*” OR “student*”)
30	“advance* notice” AND (“review” OR “meta-analy*”)
31	“school climate” AND (“systematic review” OR “meta-analy*”)
32	SU “routines” AND (“parent*” OR “famil*” OR “classroom*” OR “school*” OR “child*” OR “adolescen*” OR “teen*” OR “youth” OR “student*”) AND (“review” OR “meta-analy*”)
33	“activity schedule*” AND (“review” OR “meta-analy*”)
34	SU “rules” AND (“parent*” OR “famil*” OR “classroom*” OR “school*” OR “child*” OR “adolescen*” OR “teen*” OR “youth” OR “student*”) AND (“systematic review” OR “meta-analy*”)

35	SU (“policies”) AND (“disciplin*” OR “behavio*”) AND (“parent*” OR “famil*” OR “classroom*” OR “school*” OR “child*” OR “adolescen*” OR “teen*” OR “youth” OR “student*”) AND (“review” OR “meta-analy*”)
36	“social script*” AND (“review” OR “meta-analy*”)
37	“script fading” AND (“review” OR “meta-analy*”)
38	SU “ritual*” AND (“parent*” OR “famil*” OR “classroom*” OR “school*”) AND (“review” OR “meta-analy*”)
39	“Noncontingent reinforcement” AND (“review” OR “meta-analy*”)
40	SU “story telling” AND “behavio*” AND (“review” OR “meta-analy*”)
41	“social stor*” AND (“review” OR “meta –analy*”)
42	(“distraction” NOT (“osteogenesis” OR “mandibular”)) AND (“parent*” OR “famil*” OR “classroom*” OR “school*” OR “child*" OR "adolescen*" OR “teen*” OR “youth” OR “student*”) AND (“systematic review” OR “meta –analysis”)
43	“redirection” and “behavio*” AND (“review” OR “meta-analy*”)
44	SU_“Praise” AND (“parent*” OR “famil*” OR “classroom*” OR “school*” OR “child*" OR "adolescen*" OR “teen*” OR “youth” OR “student*”) AND (“review” OR “meta –analy*”)
45	SU (“reward*” OR “incentiv*”) AND (“disciplin*” OR “behavio*”) AND (“parent*” OR “famil*” OR “classroom*” OR “school*” OR “child*” OR “adolescen*” OR “teen*” OR “youth” OR “student*”) AND (“systematic review” OR “meta –analy*”)
46	“reinforcement” AND (“parent*” OR “famil*” OR “classroom*” OR “school*” OR “child*” OR “adolescen*” OR “teen*” OR “youth” OR “student*”) AND (“systematic review” OR “meta-analy*”)
47	“contingenc*” AND (“disciplin*” OR “behavio*”) AND (“parent*” OR “famil*” OR “classroom*” OR “school*” OR “child*” OR “adolescen*” OR “teen*” OR “youth” OR “student*”) AND (“systematic review” OR “meta-analy*”)
48	“contingency contract*” AND (“review” OR “meta-analy*”)
49	“contingent music” AND (“review” OR “meta-analy*”)
50	“good behavio* game” AND (“review” OR “meta-analy*”)
51	“extinction” AND “behavio*” AND (“systematic review” or “meta-analy*”)
52	SU ("fading" AND "behavio*") AND (“review” or “meta-analy*”)
53	SU (“systematic desensitization”) AND (“parent*” OR “famil*” OR “classroom*” OR “school*” OR “child*” OR “adolescen*” OR “teen*” OR “youth” OR “student*”) AND (“review” or “meta-analy*”)
54	“chaining” AND “behavio*” AND (“review” OR “meta-analy*”)
55	“graduated exposure” and (“review” OR “meta-analy*”)
56	("Time-out" OR "Timeout") AND (“parent*” OR “famil*” OR “classroom*” OR “school*” OR “child*” OR “adolescen*” OR “teen*” OR “youth” OR “student*”) AND (“review” or “meta-analy*”)
57	(“token economy” OR “response cost”) AND (“review” or “meta-analy*”)
58	SU “behavio* contract*” AND (“review” OR “meta-analy*”)
59	“family meeting*” AND (“review” OR “meta-analy*”)
60	“family conferenc*” AND (“review” OR “meta-analy*”)
61	“collaborative problem-solving” AND (“parent*” OR “famil*” OR “classroom*” OR “school*” OR “child*” OR “adolescen*” OR “teen*” OR “youth” OR “student*”) AND (“review” OR “meta-analy*”)

62	SU "problem solv*" AND ("disciplin*" OR "behavio*") AND ("parent*" OR "famil*" OR "classroom*" OR "school*" OR "child*" OR "adolescen*" OR "teen*" OR "youth" OR "student*") AND ("systematic review" OR "meta-analy*")
63	"restitution" AND ("systematic review" or "meta-analy*")
64	"circle*" AND "restorative" AND ("review" OR "meta-analy*")
65	("victim offender conferenc*" OR "victim offender mediation") AND ("systematic review" OR "meta-analy*")
66	"family group conferenc*" AND ("systematic review" OR "meta-analy*")
67	"restorative" AND "conferenc*" AND ("systematic review" OR "meta-analy*")
68	"restorative justice" AND ("systematic review" OR "meta-analy*")
69	SU ("crisis intervention" AND ("parent*" OR "famil*" OR "classroom*" OR "school*" OR "child*" OR "adolescen*" OR "teen*" OR "youth" OR "student*") AND ("review" OR "meta-analy*"))
70	"crisis intervention" AND ("violen*" OR "aggress*") AND ("parent*" OR "famil*" OR "classroom*" OR "school*" OR "child*" OR "adolescen*" OR "teen*" OR "youth" OR "student*") AND ("review" OR "meta-analy*")
71	SU (("emotion*" OR "affect") AND "regulation" AND ("parent*" OR "famil*" OR "classroom*" OR "school*" OR "child*" OR "adolescen*" OR "teen*" OR "youth" OR "student*")) AND ("review" OR "meta-analy*")
72	("de-escalation" AND ("review" OR "meta-analy*")) NOT ("microbial*" OR "antibiotic*" OR "antimicrobial*" OR "virus" OR "carcinoma" OR "cancer" OR "fungal" OR "disease")
73	"tantrum*" AND ("review" OR "meta-analy*")
74	"containment" AND "aggressi*" AND ("parent*" OR "famil*" OR "classroom*" OR "school*" OR "child*" OR "adolescen*" OR "teen*" OR "youth" OR "student*") AND ("review" OR "meta-analy*")
75	"daily report" AND ("review" OR "meta-analy*")
76	SU ("goal setting") AND ("parent*" OR "famil*" OR "classroom*" OR "school*" OR "child*" OR "adolescen*" OR "teen*" OR "youth" OR "student*") AND ("review" or "meta-analy*")
77	"self-regulation" AND ("disciplin*" OR "behavio*") AND ("parent*" OR "famil*" OR "classroom*" OR "school*" OR "child*" OR "adolescen*" OR "teen*" OR "youth" OR "student*") AND ("systematic review" or "meta-analy*")
78	("high probability command sequence") AND ("review" OR "meta-analy*")
79	"Reprimand" AND ("disciplin*" OR "behavio*") AND ("parent*" OR "famil*" OR "classroom*" OR "school*" OR "child*" OR "adolescen*" OR "teen*" OR "youth" OR "student*") AND ("review" or "meta-analy*")
80	"fixed-time reinforcement" AND ("review" OR "meta-analy*")
81	"seclusion" AND ("parent*" OR "famil*" OR "classroom*" OR "school*" OR "child*" OR "adolescen*" OR "teen*" OR "youth" OR "student*") AND ("review" or "meta-analy*")
82	"Student participation" AND ("disciplin*" OR "behavio*") AND ("review" or "meta-analy*")

References

1. Quail, K.R.; Ward, C.L. Nonviolent discipline options for caregivers and teachers: a systematic overview of the evidence. *Trauma, Violence, & Abuse*. **2022,** *23,* 620-638. https://doi.org/10.1177/1524838020967340
2. Becker, L.A.; Oxman, A.D. Overviews of reviews. In *Cochrane Handbook for Systematic Reviews of Interventions*; Higgins, J.P.T., Green, S., Eds.; Cochrane book series; John Wiley & Sons, Ltd.: Chichester, England, 2008, pp. 607–631. https://doi.org/10.1002/9780470712184.ch22.
3. Pollock, M.; Fernandes, R.M.; Becker, L.A.; Featherstone, R.; Hartling, L. What guidance is available for researchers conducting overviews of reviews of healthcare interventions? A scoping review and qualitative metasummary. *Systematic Reviews,* **2016**, *5*(1), 190-204. https://doi.org/10.1186/s13643-016-0367-5
4. Thomson, D.; Foisy, M.; Hartling, L. (Producer). Overviews of reviews [Recording of Cochrane Canada Webinar] **2013**. Retrieved from training.cochrane.org/resource/overviews-reviews (accessed on 1 February 2018).
5. Wright, J.; Walwyn, R. Literature search methods for an overview of reviews. ('umbrella' reviews or 'review of reviews'). **2016**. Retrieved from (https://medhealth.leeds.ac.uk/.../literature_search_methods_for_overviews_of_reviews) (accessed on 1 February 2018).
6. Pollock, M.; Fernandes, R.M.; Hartling, L. Evaluation of AMSTAR to assess the methodological quality of systematic reviews in overviews of reviews of healthcare interventions. *BMC Med Res Methodol,* **2017**, *17*(1), 48-60. doi:10.1186/s12874-017-0325-5
