# Supplementary material for: Using Non-Violent Discipline Tools: Evidence Suggesting the Importance of Attunement
Source: Int J Environ Res Public Health. 2023 Dec 15;20(24):7187. doi: 10.3390/ijerph20247187 (PMC10742770; doi:10.3390/ijerph20247187)
Supplement: Supplementary file 1 [file ijerph-20-07187-s001.zip › File S4 - Attuned discipline in practice.pdf]

# Supplement 4: Attuned discipline in practice

## 1. Introduction

This supplement presents everyday examples, to demonstrate attunement as it could apply to practice of discipline by caregivers and teachers. Three important areas will be discussed here: understanding when and where a child needs behavioral support; choosing tools that would best fit the situation and needs of the child; and understanding when to stop intervening or to fade the support.

## 2. Understanding when and where a child needs behavioral support

The concept now widely referred to as scaffolding [1] comes from the Vygotskian concept of the zone of proximal development (ZPD) [2] (figure 1). At any stage of development, there are things the child cannot do, things they can do, and things they can only do with some support. This last category is their ZPD [2].

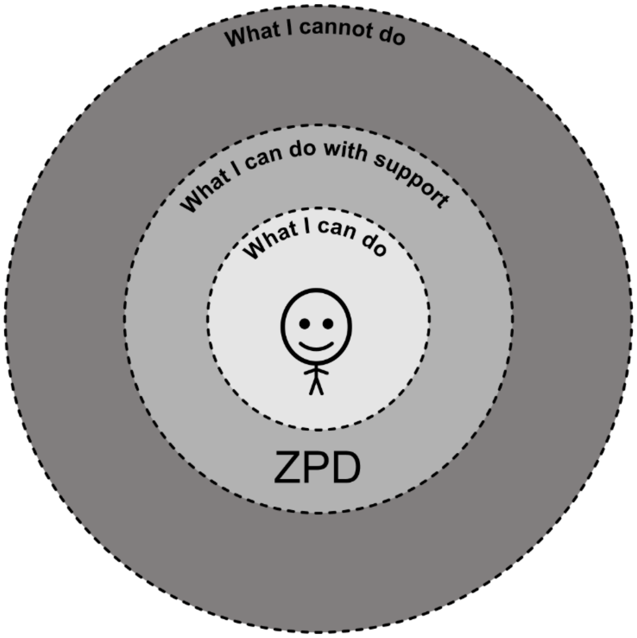


**Figure 1:** The zone of proximal development (ZPD)

The role of the caregiver or teacher is to scaffold the child's learning in the zone of proximal development, providing support when it is needed and fading that support as they begin to manage

something on their own [3]. In this way, the caregiver or teacher supports the child to manage something just beyond what they would be able to manage unassisted, with the idea that, with that assistance, they will develop the competence to manage by themselves [2]. Our focus here, is on child self-regulation and appropriate behaviour as the specific areas of learning in the ZPD.

From this perspective, discipline is conceptualized as support rather than punishment, and includes addressing misbehaviour and teaching appropriate behaviour as forms of scaffolding. Attunement in discipline would include noticing when and where a child needs support to behave better, appropriate fit between tools used and the kind of support the child needs, and accurate matching of the level of adult support to the child's level of competence and emerging self-regulation, adding support when it is needed, and fading it when it is no longer needed. Another way of looking at this would be to say that attunement in discipline requires the adult to be both responsive and appropriately demanding of the child in the balance which best supports their eventual autonomy [4-6]. Misattunement in this context would include overinvolvement of the adult in the areas a child can manage on their own (such as overprotection, excessive direction, or rewarding a child who is already intrinsically motivated to do something), a lack of support in an area the child needs it (such as a lack of boundary, or not providing any extra support for a child with ADHD), or trying to scaffold the development of a competence the child would not be able to manage at that age or stage (such as trying to toilet train a child not developmentally ready for toilet training).

Returning to the zone of proximal development (ZPD), the solid line in figure 2 and 3 indicates adult involvement using limits and discipline tools. The area inside the solid line is therefore the amount of freedom and responsibility the child is allowed. An important part of attuned discipline would be adjusting this line so that it falls in the zone of proximal development.

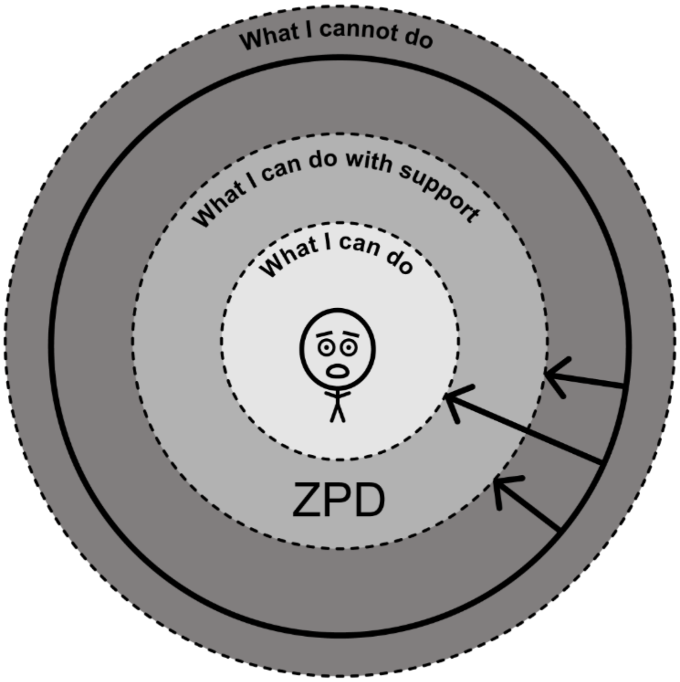


**Figure 2:** Too much freedom & responsibility

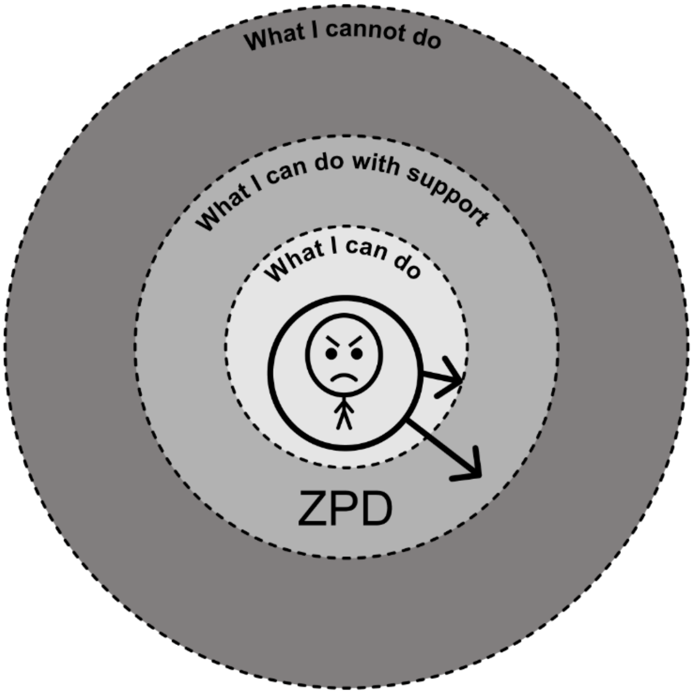


**Figure 3:** Too little freedom & responsibility

If the child is given too much freedom (figure 2), in other words they are allowed or expected to manage without adult support in the outer circle, they will not be able to manage this freedom and responsibility and there will be negative effects. Examples would include a young child with unsupervised access to a cellular phone, who starts looking at pornographic material, or a young child left at home unsupervised, who injures themselves. In each of these situations it would be unfair for the caregiver to be angry with the child, because the problematic behavior was the outcome of the child being allowed freedom and responsibility that they were not developmentally ready to manage. When a child is in this situation, it is the responsibility of the adult to reduce the child’s freedom and responsibility until they are mature enough to manage more, moving the solid line back to the ZPD, to the edge of what the child can manage.

In this outer zone, what is often needed from caregivers is protection. For example, if the child cannot yet swim, the adult should not leave them unsupervised near a swimming pool. Another example would be the important gatekeeping role caregivers have in limiting availability of things that would be bad for the child, such as excessive screen time or inappropriate media content [7], alcohol [8-10], or unhealthy food [11-14]. Recognizing that appropriate self-regulation in these areas falls in the zone of things the child cannot yet manage, the parents take on the responsibility, protectively, to regulate and limit access to these things. These monitoring or restrictive actions are attuned to the developmental level of the child. Not offering protection where it is needed would be misattunement, and a form of neglect.

If the solid line falls in the area the child can manage (figure 3), representing adult over-involvement or overcontrol, the adult would need to adjust in the other direction, to back off, allowing the child the responsibility and freedom they are ready for. In this area the child needs opportunities to exercise autonomy and self-regulation. Adult over-involvement, such as directing the child unnecessarily, or over-involving themselves in the child's schoolwork would be intrusive, and an act of misattunement.

The solid line (adult involvement) should fall at the edge of what the child can manage, in the ZPD, where the child needs adult support, and therefore where it is helpful to scaffold using discipline tools. Examples would include using a daily report card for a child with ADHD to help them exercise more self-regulation in class or using a reward system to motivate children to do certain tasks at home.

To understand when and where a child needs behavioral support, the adult could use tools that are likely to increase attunement, such as monitoring and listening. If the child is managing well, the adult would not need to scaffold further except perhaps with praise. If the child is not managing with something, the adult would need to attune further to whether the child cannot be expected to manage that thing developmentally, or whether they may be able to manage with support. In the former case,

more limits and protection may be needed. In the latter case it would be appropriate to scaffold using discipline tools.

**3. Choosing tools that best fit the situation and needs of the child**

Not only do children need adult support in the ZPD, they need the right kind of support, to learn to manage on their own in future. Here the adult needs to use attunement in the form of choosing supportive interventions that fit best with the child's needs and evaluating whether they are having the desired effect, or whether a different supportive skill should be used. Here are some real examples of scaffolding using discipline tools in the zone of proximal development. Names have been changed.

**3.1.** A teacher is supervising a group of young children, 3 to 6 years old, in a play area. The fact that she is supervising them is already a form of scaffolding. They are too young to be expected to play safely on their own, but manage well with her there. The fact that there are rules is also a form of scaffolding. One of the rules is that the children may not go out of bounds. The teacher has shown them where they may or may not play. Occasionally the younger children seem to forget the boundaries and wander over into an out-of-bounds area. The teacher prompts them, verbally, to return to the play area. This reminder is enough scaffolding for most of the children and they return to play happily within bounds.

Four-year-old Mila walks over the boundary. The teacher sees this and prompts her with a reminder of where she may and may not go. Since, in this case, Mila is being defiant and not forgetful, this first attempt at scaffolding better behavior fails. Mila turns to look at the teacher, then turns back and marches purposefully to a swing in another play area. The teacher uses another skill from her toolkit of possible interventions, a timeout. Since there is no aggression, Mila does not need an exclusionary timeout. She is managing being with other people, but she is not managing the outside rules, so the teacher gives her a brief timeout from the thing she is not managing, which is playing outside.

The teacher leads her inside and says: “You are going to play inside now.” “But I want to play outside,” Mila says. “I know,” says the teacher in an understanding voice, “you love playing outside,” (here the teacher uses an active listening-type reflection to support Mila through the disciplinary process), “but we tried that, and it did not go well. You need to play inside.” Mila looks at the teacher: “I won’t go where I’m not supposed to go,” she says. “We tried playing outside, and you did go where you were not supposed to go,” says the teacher, “so now you must play inside. If you manage that nicely, then, in a little while, I will give you another chance to play outside.” Mila plays inside, interacting with other children who are playing there. After 5 minutes the teacher asks: “If I let you go outside now, do you think you will manage the outside rules? “Yes!” says Mila. “And you won’t go where you are not supposed to go?” “No!” “All right, let’s see how it goes: you may play outside.” Mila plays very nicely and does not go out of bounds that day or any other day thereafter.

Note the attunement in the use of timeout: the teacher matches the kind of timeout given to what Mila is not managing. This is an example of a non-exclusionary time-out. Experiencing a sudden reduction of her freedom, and realizing that getting it back is conditional on her staying in bounds, provides the motivation needed for Mila to co-operate with the outside rules. The trial-and-error process in which the teacher first uses a prompt and then a timeout to address the child’s behavior is part of the process of attunement, rather than a misattunement. Checking whether the prompt works helps the teacher diagnose that the child is being defiant and needs a different intervention. Using a time-out immediately would have been unnecessary for the other children, who only needed prompts. A principle of minimum intervention makes sense in the ZPD, because the less the adult does, the more self-regulation the child has the opportunity to exercise.

**3.2.** Jared, 5, is very angry because his parents have said no to something he wanted to do. He nags and nags for what he wants, but his parents stand firm. His mother tries to show understanding by using active listening and reflecting his disappointment at her answer. Sometimes this calms him, but this time Jared does not respond to active listening and does not allow her to comfort him. He screams

and starts hitting her. Mom immediately puts him in his room for a 3-minute time-out. Jared knows about timeouts as his parents use them consistently for any kind of aggression. They have explained this to him, saying: “Sometimes we need to stop you, but one day you will be able to stop yourself.” After the timeout Jared is still upset, but no longer being aggressive. He apologizes and allows his mother to comfort him with a hug and help him to understand his feelings with active listening.

As Jared gets older, he learns to take himself to timeout and runs to his room when he gets very angry, without hitting people first. After he does this, his parents praise him and tell him how proud they are of him for realizing by himself that he needed a timeout and for managing not to hit anyone. As Jared gets even older, he manages a lot of similar situations without losing his temper and without needing to go to timeout at all. Here we can see how, as Jared’s capacity for self-regulation increases, parental intervention appropriately decreases, from using timeout, to using praise, to not needing to do anything at all.

**3.3.** On the weekend, Aiden, 16, spends a lot of time playing games and watching YouTube videos on his phone, leaving his household chores undone. His parents prompt him to do the dishes, and he promises to “do them in a minute.” Two hours go by, and he does not get around to the chore. His parents use a timeout. Sending him to his room would provide further escape from the dishes, so they adjust his freedom only in the area he is not managing, and give him a timeout from his phone. As his mother asks him to hand over the phone, she adds a contingency: he can have it back when the dishes are done. Aiden grumbles, but washes the dishes immediately, then asks for his phone. His mother thanks him for washing the dishes, and returns his phone.

These three examples concern the attuned use of timeout, but there are many other behavioral skills that can be used to scaffold in the ZPD [15]. One of these is reward, which can be particularly useful when caregivers want a child to do something that the child is not at all motivated to do [16,17].

**3.4**. Six-year-old Jade is capable of dressing herself in the morning, but keeps saying “I can’t,” or “I want you to help me.” If she would dress herself it would certainly make things easier for her

parents, but where is the motivation for Jade? She enjoys the attention she receives when her mother helps her get dressed. If she dresses herself, she will be alone, while her mother helps her younger brother. Her mother recognizes that Jade’s helplessness is attention-motivated, and supports her by adding some motivation in the form of a reward involving attention. Jade dresses herself without complaint, knowing that each time she does this, she earns an extra story, read to her by her mother in the afternoon. Soon, dressing herself becomes a habit, and her mother fades out the use of the reward by shifting it to another area where Jade needs motivation.

Here we can see that the support Jade needed was added motivation. Her mother could have rewarded her with an unrelated reward, such as a sticker, but instead matched the kind of reward to the function of Jade’s behavior (attention), increasing the likelihood of success and child satisfaction by using a more attuned intervention.

The examples above focus mainly on attuned use of timeout and reward. These two skills were chosen because, aside from being commonly known, they are both somewhat controversial. Despite decades of studies showing positive outcomes associated with these tools [17-23], popular literature, internet advice and even academic articles have warned against dangers associated with their use [18,24-29]. These two controversies actually lend support to the argument for attunement in discipline.

The main concern about timeout seems to be damage to the attachment bond by communicating to the child that the parent is not available to them in times of distress [19]. This is a legitimate concern, but not supported by evidence, and there are several reasons why this damage is not only unlikely, but that appropriate use of timeout could support secure attachment [19]. A further argument is added here: If we look at examples used in texts warning against the use of timeout [25,28,29], they describe children isolated at times when they are in distress, and need soothing and comfort. This would be an example of inappropriate or misattuned use of time-out. The authors of two of the above articles have since retracted some of what they said, and confirmed that they were referring to inappropriate use of timeout [30]. From the perspective of attunement, timeout is not good or bad in itself, but should be

evaluated by its fit with the needs of the child. Attuned use of timeout, as described in the examples above, should not be problematic: after all, attachment security is built through attuned responses [31,32,33,34].

The main concern about reward is the undermining of intrinsic motivation [26,27]. We argue that this is not an indication that reward should not be used, rather that reward should be used with attunement. Research has indeed shown that if a child is already intrinsically motivated to do something, rewards could undermine that motivation [35]; however if intrinsic motivation is low, reward enhances motivation and can thus be a constructive intervention [16,35]. Praise has been found to have different effects, enhancing intrinsic motivation and self-reported interest [16,35]. Does this indicate that praise is a better skill and should be used instead of reward? Further research shows that praise is not always enough to motivate compliance [23,36]. In the case of compliance, reward has been shown to be more effective [23]. The best option for caregivers is thus to use rewards or praise with attunement to the motivation level of the child. If there is no or very low motivation to do something, it is safe to use reward [16,35], and reward is likely to work better than praise to achieve compliance [23]. If motivation is high, however, it would be safer to use praise [16,35].

If we relate this to the diagrams, praise would be safe to use in the area where a child can manage or in the ZPD. Reward would be unnecessary and unconstructive if the child is already motivated (the inner circle), but it may be very helpful in the ZPD. Neither reward nor praise would work in the outer circle, for things the child cannot manage developmentally.

**4. Understanding when to stop**

Knowing when to stop intervening can be very difficult for caregivers, many of whom may be used to judging the effectiveness of discipline by the amount of distress or suffering it causes the child [19]. Non-violent discipline tools are drawn from different fields and schools of thought [15]. Each of these offer different perspectives, from which we can draw guidance in this important aspect of attunement.

From a behavioral perspective, discipline is effective when problem behavior has been reduced or eliminated [19] or an appropriate target behavior learned. An important part of behavioral interventions is to fade support gradually once the child is coping better [37]. One would thus consider the intervention complete when it is effective, when supports such as rewards and prompts have been removed, and the child is managing the target behavior by themselves.

From a self-regulation perspective, one would look for signs that the child is able to exercise self-control, and fade adult control as this becomes apparent. More intervention than is needed would be misattuned and reduce opportunities for the child to exercise their emerging capacity for self-regulation [38].

From a collaborative problem-solving perspective [39,40], a discipline problem would be considered resolved when child and adult agree that the solution they had agreed on collaboratively, and implemented, was successful. If not, they would discuss further and try another solution until they found one that works.

From an attachment perspective, misattunements and periodic disconnection are normal parts of attachment relationships. What is important is that when there is disconnection, which may happen around challenging behavior, there should, as soon as possible, be repair [41]. Thus, from an attachment perspective, a discipline problem would only be considered resolved when caregiver and child have reconnected.

From a restorative justice perspective, one would look beyond the individual child to the others affected by the problem behavior. From this perspective, problem behavior occurs in the context of relationship, and incurs responsibility to repair or engage in restitution for any damage done. Discipline would be defined as an attempt to repair the harm a problem behavior has caused, rather than inflicting harm on the offender [42]. From this perspective one could stop intervening when appropriate reparation has been made to those affected by the problem behavior.

With the guidance of these different perspectives, caregivers could ask themselves questions to aid their attunement at the end part of intervening, such as: Has the problem been solved, or problem behavior ceased? Is the child now engaging in the desired behavior? Is there any unfinished business such as fading supports, apologizing, reconnecting, or making amends?

## 5. Conclusion

In this supplement, real examples were used to illustrate the practice of attuned discipline. Three important areas were highlighted: understanding when and where a child needs behavioral support; choosing tools that would best fit the situation and needs of the child; and understanding when to stop intervening or to fade support. It is hoped that this will give practical guidance to readers wanting to use non-violent discipline tools with attunement.

## References


1. Berk, L. E., & Winsler, A. (1995). Scaffolding children's learning: Vygotsky and early childhood education. *NAEYC Research into Practice Series.* Volume 7. Washington DC: National Association for the Education of Young Children. https://eric.ed.gov/?id=ed384443
2. Vygotsky, L. (1978). Interaction between learning and development. *Readings on the development of children, 23*(3), 34-41.
3. Li, J., & Julian, M. M. (2012). Developmental relationships as the active ingredient: A unifying working hypothesis of "what works" across intervention settings. *American Journal of Orthopsychiatry, 82*(2), 157. https://doi.org/10.1111/j.1939-0025.2012.01151.x
4. Baumrind, D. (1966). Effects of authoritative parental control on child behaviour. *Child Development*, 887-907. https://doi.org/10.2307/1126611
5. Baumrind, D. (1967). Child care practices anteceding three patterns of preschool behaviour. *Genetic Psychology Monographs*. Retrieved from https://www.worldcat.org/title/genetic-psychology-monographs/oclc/1440765
6. Baumrind, D. (2013). Authoritative parenting revisited: History and current status. In R. E. Larzelere, A. S. Morris, & A. W. Harrist (Eds.), Authoritative parenting: Synthesizing nurturance and discipline for optimal child development. *American Psychological Association.*, 11-34. https://doi.org/10.1037/13948-002
7. Collier, K. M., Coyne, S. M., Rasmussen, E. E., Hawkins, A. J., Padilla-Walker, L. M., Erickson, S. E., & Memmott-Elison, M. K. (2016). Does parental mediation of media influence child outcomes? A meta-analysis on media time, aggression, substance use, and sexual behavior. *Developmental Psychology, 52*(5), 798-812. doi:10.1037/dev000010

8. Kaynak, Ö., Winters, K. C., Cacciola, J., Kirby, K. C., & Arria, A. M. (2014). Providing alcohol for underage youth: What messages should we be sending parents? *Journal of Studies on Alcohol and Drugs, 75*(4), 590-605. https://doi.org/10.15288/jsad.2014.75.590
9. Ryan, S. M., Jorm, A. F., & Lubman, D. I. (2010). Parenting factors associated with reduced adolescent alcohol use: a systematic review of longitudinal studies. *Australian and New Zealand Journal of Psychiatry, 44*(9), 774-783. https://doi.org/10.1080/00048674.2010.501759
10. Sharmin, S., Kypri, K., Khanam, M., Wadolowski, M., Bruno, R., & Mattick, R. (2017). Parental supply of alcohol in childhood and risky drinking in adolescence: Systematic review and meta-analysis. *International journal of environmental research and public health, 14*(3), 287. https://doi.org/10.3390/ijerph14030287
11. Mazarello Paes, V., Hesketh, K., O'Malley, C., Moore, H., Summerbell, C., Griffin, S., . . . Lakshman, R. (2015). Determinants of sugar-sweetened beverage consumption in young children: A systematic review. *Obesity Reviews, 16*(11), 903-913. doi:10.1111/obr.12310
12. McClain, A. D., Chappuis, C., Nguyen-Rodriguez, S. T., Yaroch, A. L., & Spruijt-Metz, D. (2009). Psychosocial correlates of eating behavior in children and adolescents: A review. *International Journal of Behavioral Nutrition and Physical Activity, 6*(1), 54. https://doi.org/10.1186/1479-5868-6-54
13. Shepherd, J., Harden, A., Rees, R., Brunton, G., Garcia, J., Oliver, S., & Oakley, A. (2006). Young people and healthy eating: A systematic review of research on barriers and facilitators. *Health Education Research, 21*(2), 239-257. https://doi.org/10.1093/her/cyh060
14. Yee, A. Z. H., Lwin, M. O., & Ho, S. S. (2017). The influence of parental practices on child promotive and preventive food consumption behaviors: a systematic review and meta-analysis. *International Journal of Behavioral Nutrition & Physical Activity, 14*, 1-14. doi:10.1186/s12966-017-0501-3
15. Quail, K. R., & Ward, C. L. (2022). Nonviolent discipline options for caregivers and teachers: a

systematic overview of the evidence. *Trauma, Violence, & Abuse, 23*(2), 620-638. https://doi.org/10.1177/1524838020967340

16. Cameron, J., Banko, K. M., & Pierce, W. D. (2001). Pervasive negative effects of rewards on intrinsic motivation: The myth continues. *The Behavior Analyst, 24*(1), 1-44. https://doi.org/10.1007/bf03392017
17. Luman, M., Oosterlaan, J., & Sergeant, J. A. (2005). The impact of reinforcement contingencies on AD/HD: A review and theoretical appraisal. *Clinical Psychology Review, 25*(2), 183-213. doi:10.1016/j.cpr.2004.11.001
18. Corralejo, S. M., Jensen, S. A., Greathouse, A. D., & Ward, L. E. (2018). Parameters of Time-out: Research Update and Comparison to Parenting Programs, Books, and Online Recommendations. *Behavior Therapy, 49*(1), 99-112. doi:10.1016/j.beth.2017.09.005
19. Dadds, M. R., & Tully, L. A. (2019). What is it to discipline a child: What should it be? A reanalysis of time-out from the perspective of child mental health, attachment, and trauma. *American Psychologist*. https://doi.org/10.1037/amp0000449
20. Embry, D. D., & Biglan, A. (2008). Evidence-based Kernels: Fundamental Units of Behavioral Influence. *Clinical Child and Family Psychology Review, 11*(3), 75-113. doi:10.1007/s10567-008-0036-x
21. Kaminski, J. W., Valle, L. A., Filene, J. H., & Boyle, C. L. (2008). A Meta-Analytic Review of Components Associated with Parent Training Program Effectiveness. *Journal of Abnormal Child Psychology, 36*(4), 567-589. doi:10.1007/s10802-007-9201-9
22. Ma, I., van Duijvenvoorde, A., & Scheres, A. (2016). The interaction between reinforcement and inhibitory control in ADHD: A review and research guidelines. *Clinical Psychology Review, 44,* 94-111. doi:10.1016/j.cpr.2016.01.001

23. Owen, D., Slep, A., & Heyman, R. (2012). The Effect of Praise, Positive Nonverbal Response, Reprimand, and Negative Nonverbal Response on Child Compliance: A Systematic Review. *Clinical Child & Family Psychology Review, 15*(4), 364-385. doi:10.1007/s10567-012-0120-0

24. Drayton, A. K., Andersen, M. N., Knight, R. M., Felt, B. T., Fredericks, E. M., & Dore-Stites, D. J. (2014). Internet guidance on time out: Inaccuracies, omissions, and what to tell parents instead. *Journal of Developmental and Behavioral Pediatrics, 35*(4), 239-246. doi:10.1097/DBP.0000000000000059

25. Durrant, J. E., & Stewart-Tufescu, A. (2017). What is "Discipline" in the Age of Children's Rights? *The International Journal of Children's Rights, 25*(2), 359-379. doi:10.1163/15718182-02502007

26. Kohn, A. (1999). *Punished by rewards: The trouble with gold stars, incentive plans, A's, praise, and other bribes*: Houghton Mifflin Harcourt. ISBN 978-0-618-00181-1

27. Kohn, A. (2003). Almost There, but Not Quite. *Educational Leadership, 60*(6), 26-29. Retrieved from http://www.ascd.org/publications/educational-leadership.aspx

28. Siegel, D., & Payne Bryson, T. (2014a). Time-outs' are hurting your child. *Time, September, 23*. Retrieved from http://time.com/3404701/disciplinetime-out-is-not-good

29. Siegel, D. J., & Payne Bryson, T. (2014b). The trouble with time-outs. *Scholastic Parent & Child,, 22*(2), 40-45. Retrieved from http://www.scholastic.com/earlylearner/parentandchild/publix/

30. Siegel, D., & Payne Bryson, T. (2014c). You said WHAT about time-outs. *Huffington Post*. Retrieved from https://www.huffington post.com/daniel-j-siegel-md/time-outs-overused_b_6006332.html

31. Ainsworth, M., Blehar, M., Waters, E., & Wall, S. (1978). Patterns of attachment: A psychological study of the strange situation. *Child Development, 41*, 49-67. https://doi.org/10.4324/9780203758045

32. Ainsworth, M. D. (1967). *Infancy in Uganda: Infant care and the growth of love*. Baltimore: Johns Hopkins Press. https://doi.org/10.1542/peds.41.3.700a

33. Isabella, R. A., & Belsky, J. (1991). Interactional synchrony and the origins of infant-mother attachment: A replication study. *Child Development, 62*(2), 373-384. https://doi.org/10.2307/1131010

34. Isabella, R. A., Belsky, J., & von Eye, A. (1989). Origins of infant-mother attachment: An examination of interactional synchrony during the infant's first year. *Developmental Psychology, 25*(1), 12. https://doi.org/10.1037/0012-1649.25.1.12

35. Deci, E. L., Koestner, R., & Ryan, R. M. (1999). A meta-analytic review of experiments examining the effects of extrinsic rewards on intrinsic motivation. *Psychological Bulletin, 125*(6), 627-668. doi:10.1037/0033-2909.125.6.627

36. Leijten, P., Gardner, F., Melendez-Torres, G. J., Knerr, W., & Overbeek, G. (2018). Parenting behaviors that shape child compliance: A multilevel meta-analysis. *PLoS ONE, 13*(10), 1-15. doi:10.1371/journal.pone.0204929

37. Carlson, J., & Mayer, G. R. (1971). Fading: A behavioral procedure to increase independent behavior. *The School Counselor, 18*(3), 193-197. Retrieved from https://www.jstor.org/stable/23896513

38. Troutman, B. (2015). *Integrating behaviorism and attachment theory in parent coaching.* : Springer. https://doi.org/10.1007/978-3-319-15239-4

39. Greene, R. W., & Ablon, J. S. (2005). *Treating explosive kids: The collaborative problem-solving approach*: Guilford Press. https://doi.org/10.1177/0734016809332179

40. Pollastri, A. R., Epstein, L. D., Heath, G. H., & Ablon, J. S. (2013). The collaborative problem solving approach: Outcomes across settings. *Harvard Review of Psychiatry, 21*(4), 188-199. doi: 10.1097/HRP.0b013e3182961017

41. Karen, R. (1994). *Becoming attached: First relationships and how they shape our capacity to love*: Oxford University Press, USA. ISBN 978-0-19-511501-7

42. Strang, H., Sherman, L. W., Mayo-Wilson, E., Woods, D., & Ariel, B. (2013). Restorative Justice Conferencing (RJC) Using Face-to-Face Meetings of Offenders and Victims: Effects on Offender Recidivism and Victim Satisfaction. A Systematic Review. *Campbell Systematic Reviews*. https://doi.org/10.4073/csr.2013.12
