# Supplementary figures and images for: Using Non-Violent Discipline Tools: Evidence Suggesting the Importance of Attunement
Source: Int J Environ Res Public Health. 2023 Dec 15;20(24):7187. doi: 10.3390/ijerph20247187 (PMC10742770; doi:10.3390/ijerph20247187)

## Supplement 2: PRISMA Flow Diagram

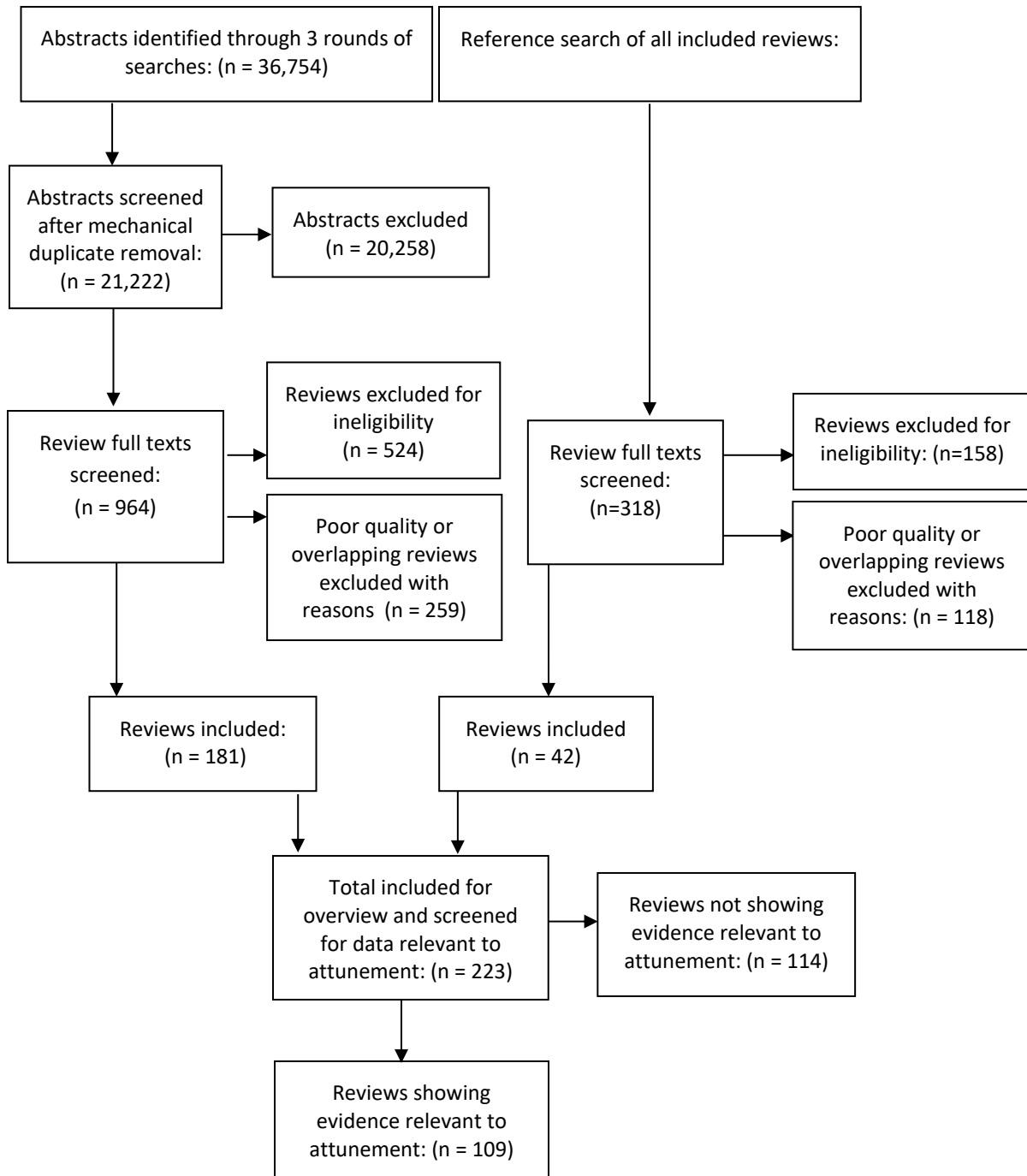

Supplement: Supplementary file 1 [file ijerph-20-07187-s001.zip › File S2 - PRISMA Flow Diagram.pdf]
